# Investigating the factors affecting farmers’ intention to adopt contract farming

**DOI:** 10.1038/s41598-024-60317-x

**Published:** 2024-04-27

**Authors:** Fatemeh Khalili, Shahla Choobchian, Enayat Abbasi

**Affiliations:** Department of Agricultural Extension and Education, College of Agriculture, Tarbiat Modares University (TMU), Tehran, 1497713111 Iran

**Keywords:** Perceived ease of use, Attitude, Trust, Awareness, Sustainable development, Sustainability, Environmental social sciences

## Abstract

Agricultural endeavors, especially in developing nations, entail inherent risks. Amidst challenges related to capital and agricultural marketing, contract farming emerges as a highly effective strategy. It not only facilitates capital accumulation but also ensures consistent product sales, establishes fair pricing, and contributes to the overall balanced development of the agricultural sector. This concern has been a longstanding global consideration, with Iran now addressing it. Recognizing the paramount importance of implementing contemporary agricultural methodologies, including contract farming, this research systematically investigates factors influencing farmers’ intentions in Iran. A survey methodology is employed for systematic information collection from a statistical population of 98,777 farmers in rural Markazi Province, Iran. Using the Karjesi and Morgan table for sample size determination, a representative subset of 383 farmers is selected through stratified random sampling, ensuring proportional assignment within strata. A researcher-made questionnaire, validated by expert panels and confirmed for reliability through Cronbach’s alpha coefficient, serves as the research instrument. Data analysis was conducted using SPSS 27, and structural equation modeling was performed with SmartPLS4. The findings reveal that trust (0.528), awareness (0.332), and attitude (0.168), exert the most substantial causal influence on farmers’ intention to embrace contract farming. Consequently, the research findings offer practical recommendations for the adoption of contract farming, providing valuable insights to policymakers and stakeholders for implementing targeted interventions aimed at boosting farmers’ willingness to participate in contractual agreements.

## Introduction

Changes in population and income, as well as advancements in technological processes at both the macroeconomic level and within food systems, are poised to impact poverty, inequality, and food security across various dimensions. Notably, poverty remains concentrated in rural areas, and due to persistent inequality, achieving the goal of eradicating hunger by 2030, as outlined by the FAO in 2017, appears challenging based on current trends^1^.

Recognizing the intricate connections between food security, nutrition, human health, ecosystem viability, climate change, and social justice, sustainable development necessitates resilient food systems. This perspective is emphasized by Caron^2^, underscoring the critical role of food systems in achieving sustainable development goals.

Sustainable agricultural intensification emerges as a pivotal strategy for land conservation, as highlighted by the FAO in 2018. Agronomic expansion is identified as one of the most effective instruments to alleviate widespread poverty, promote shared prosperity, and accommodate the projected population of 9.7 billion individuals by 2050. The agricultural sector’s evolution proves two to four times more efficient in enhancing profits for the most vulnerable compared to other sectors.

Furthermore, farming plays a fundamental role in economic growth, contributing to 4% of the world’s gross domestic product. In many developing regions, agriculture can account for more than 25% of GDP, showcasing its significance in driving economic development^3^. According to the FAO in 2023, the global agriculture value added reached 3.7 trillion in 2021. These figures underscore the substantial economic impact and potential for positive change within the agriculture sector on a global scale^4^. The progress driven by farming, the reduction of deprivation, and the availability of food face significant threats. Numerous shocks, ranging from disruptions associated with the coronavirus to extreme climate events and conflicts, are impacting food systems. These disruptions lead to higher food prices and an increase in malnutrition. Wars, in particular, have triggered global food crises, pushing millions of people into severe deprivation^3^.

The repercussions of these vulnerabilities also contribute to income instability. Agriculture bears a disproportionate share of the impact of natural disasters, with many of these challenges directly affecting smallholders^1^. In light of the persistence of these crises, countries and societies must adopt more effective and sustainable strategies to build resilience and capacity to cope with shocks and stressors^1^.

Examining the evolution of government policies in agriculture reveals that developed countries and some developing nations, acknowledging the inefficiency of government intervention policies, have shifted towards utilizing market-based tools. Financial instruments, including certificates of deposit, commodity funds, and agricultural contracts, have emerged as a result of changes in factors influencing the supply and demand of agricultural products^5^.

Contract farming (CF) is among the approaches that can effectively address the drawbacks of the agronomic sector within the framework of market dynamics^6^. At the heart of CF lies a contractual agreement between farmers and consumers, mutually establishing regulations and conditions for the production and sale of cultivated items. These terms typically outline the remuneration for producers, the quantity and quality of items requested by buyers, and the delivery date. In certain situations, the agreement may also include additional detailed information on the production process or the agricultural inputs provided by the customer^4^.

In the 1970s, Contract Farming (CF) emerged as a pro-poverty-alleviation approach in agricultural and rural development^7^. CF involves an agreement between farmers and processing/marketing companies for agricultural goods production and supply under specific contracts, often at predetermined prices^8^. Supporting institutions provide multifaceted functions such as risk management, capital and credit provision, information exchange, technology development, and legal services, enhancing added value production within the agricultural sector’s chain^10^. Extensive literature documents CF’s impact on farmers’ productivity, efficiency, and income in both developed and developing countries^7,9^, establishing a meaningful farm-to-market connection. CF is attractive for planners fostering rural development, facilitating economically disadvantaged rural participation in industrial crop cultivation for access to commerce benefits through value-added processes^10,11^.

Transitioning to a market-oriented approach, as emphasized by Khosravi^12^, addresses market needs. Farmers adopting a market-oriented approach align their production plans with market signals, focusing on cultivating more marketable commodities^13^. Agricultural commercialization, based on market orientation, is imperative for breaking free from the poverty trap, especially in developing countries, requiring active encouragement. Market orientation significantly influences production and commercialization decisions, with specific crop choices signaling intended purposes^14^. Stabilization measures and mechanisms like commodity exchanges and contract systems support production and supply chain management, removing traditional tools and ensuring stability and growth of investments in the agricultural sector^8^.

Challenges in the agricultural sector include market intermediaries, consumer needs understanding, inadequate access to machinery, low-productivity traditional practices, insufficient capital, limited inputs access, absence of diversified industries, and ineffective cultivation models. Governmental constraints hinder effective resolution^12^. Challenges include waste in the production chain, machinery devaluation, incomplete projects, and inadequate working capital, exacerbating sector issues. In the “market” domain, deficiencies in disseminating information, planning for export/import, and diplomatic efforts pose significant issues^15^. Contract Farming (CF) emerges as an optimal agricultural model, fostering a relationship and integration between industry and agriculture for mutual benefits^16^. Within the agricultural sector of Iran, the practice of formalized contractual agreements for various transactions, encompassing diverse forms of sharing and leasing, has been pervasive over an extended period. Contract cultivation, notably in the cultivation of at least two crops, specifically cotton and sugar beet, is substantiated by documented, codified, and nearly exhaustive historical records. These records serve as valuable resources for foreign businessmen and companies engaged in operations within Iran^17^. The merits of this contractual approach include income generation^18–20^, facilitated technology transfer, risk mitigation, and assurance of product and market quality^21,22^.

The research is centered on Markazi Province, where farmers grapple with significant challenges, notably related to limited market access and the absence of a reliable pricing mechanism, confusing both farmers and buyers. Predominant issues include inadequate market access and a lack of standardized pricing references, contributing to market confusion for farmers and buyers alike. The pervasive market margin and price fluctuations nationwide result in substantial price differentials between farms and consumers, with farmers rarely realizing significant profits. Factors such as insufficient seed availability and natural risks sometimes lead to crop failure or spoilage, causing financial losses for farmers due to high harvesting costs. Challenges further encompass the prevalence of brokers, and middlemen, extensive waste in the agricultural sector, lack of production standardization, inappropriate packaging, and inadequate technical warehousing during peak harvest periods. The adoption of contract farming has witnessed a recent surge, especially in developing countries, driven by globalization and heightened demand for food products. This shift underscores the necessity for enhanced collaboration within the supply chain to ensure consumers, including hypermarkets, resorts, and hospitals, receive high-quality agricultural products that are not only safe to consume but also produced in an environmentally sustainable manner^23^. Companies specializing in the management of agricultural products are particularly drawn to contract farming as it offers a reliable source of raw materials that align with their requirements in terms of quality and quantity^1^.

It is important and necessary to know what factors affect farmers’ intention of ultimately adopting contract farming. Adopting this approach may address several issues such as immigration, employment, conversion and complementary industries, large amounts of waste, and price Fluctuation. Furthermore, it provides a guaranteed way to develop the rural community, the province, and even our country sustainably.

Understanding the factors influencing farmers’ intention to adopt contract farming is crucial, addressing issues such as migration, employment, waste reduction, and price fluctuation. Moreover, it provides a structured pathway for sustainable development at the community, provincial, and national levels.

Acceptance denotes behavior that supports and encourages the use of technology rather than inhibiting or resisting it^24^. According to Rogers and Shoemaker, adopting and utilizing a new idea is considered the optimal process for beneficial action^25^. Recently, technology adoption has become a key focus in behavioral science research. One critical aspect is comprehending why individuals either adopt or resist technology. Behavioral factors influencing the adoption of environmentally sustainable practices by farmers are categorized into three clusters: dispositional, social, and cognitive.

Dispositional factors relate to farmers’ internal tendencies to behave in specific ways. Higher adoption of sustainable practices is associated with traits such as extraversion, openness to new experiences, risk-seeking behavior, moral and environmental concern, and alignment with lifestyle farming objectives. Addressing the heterogeneity of farmers on dispositional factors can be achieved through indirect segmentation based on sociodemographic and geographic characteristics, coupled with designing appropriate mixes of mandatory and voluntary schemes.

Social factors involve farmers’ interpersonal relationships. Adoption of sustainable practices is more likely when neighboring farmers have already done so, social referents support adoption, and farmers seek social status. Promising policy options include communicating descriptive norms, focusing economic support in low-adoption areas, providing social recognition for efforts, and informing farmers of their environmental performance relative to others.

Cognitive factors pertain to learning and reasoning about sustainable practices. Adoption increases when farmers possess adequate knowledge and competencies related to these practices and perceive them as bringing environmental or financial benefits with limited risks. Policy options for addressing cognitive factors include increasing awareness of sustainable practices, framing costs and benefits appropriately to de-bias perception, and enhancing the flexibility of agri-environmental schemes^26^.

To gain a deeper understanding of farmers’ decision-making processes, it is crucial to delve into the underlying influences of their intentions. This study seeks to clarify the behavioral attitudes toward contract farming and the inclination toward its adoption. Drawing upon the framework of the technology acceptance model, in conjunction with critical factors such as awareness, trust, social impact, and external motivation, this research aims to offer comprehensive insights into farmers’ attitudes and intentions regarding participation in contract farming. These factors will be examined to illuminate the nuanced dynamics that shape farmers’ decisions in this context, thus contributing to a more thorough understanding of agricultural practices and their socio-economic implications.

## Literature review

### Contract farming

Numerous studies, both within and outside the country, have explored the concept of contract farming. Beyond a mere focus on increasing production, this agricultural method aims for broader societal, economic, and environmental benefits, encompassing employment generation, income growth, food security, and sustainable resource utilization^27^. The factors influencing farmers’ inclination toward adopting contract farming are multifaceted, involving increased income, enhanced product quality and quantity, and heightened productivity through resource conservation and unemployment reduction.

Farmers’ readiness to engage in contracts is intricately tied to the specifics of the contracts themselves, including inputs provided by companies, technical assistance, and purchasing terms. The variable pricing in contracts, coupled with farmers’ concerns about input market uncertainties, significantly influences their willingness to participate^28^. Demographic factors such as education level, ownership type, and cultivation scale also play a role in shaping farmers’ decisions to opt for contract farming. Older farmers may prefer contracts where the contracting parties play a more substantial role in input provision and crop control, particularly when dealing with factory entities, leading to improved performance, product quality, increased income, and agricultural sector development^29^.

Government contracts can positively impact farmers’ perspectives, provided they offer favorable terms such as increased product prices and supportive policies and programs. Urban and rural youth exhibit similar levels of acceptance, attitude, and awareness toward contract farming, with a higher likelihood of acceptance among the educated, young, and financially capable farmers^30^. Risk attitude becomes a pivotal factor, as farmers inclined toward risk-taking are more likely to engage in contracts^31^. Gender differences emerge, with male farmers generally trusting third-party observers more than their female counterparts when it comes to assessing product quality in potential collusion scenarios^32^.

The influence of group dynamics and agricultural extension services on contract farming participation is notable^33^. Extension training positively impacts agricultural contracts by enhancing technical skills, although resource limitations among smallholder farmers may hinder the practical application of acquired knowledge. The intention to adopt contract farming is linked to education, price volatility, credit availability, and investment capacity^34^. However, the efficacy of agricultural contracts for farmers is contingent upon the specific type of contract and its associated conditions. Furthermore, contract farming is recognized for promoting environmentally sustainable practices and potentially safeguarding ecosystems from the adverse effects of chemical fertilizers and pesticides in crop production^35,36^. Upon reviewing the literature, it was observed that there is a gap in research concerning farmers’ inclination to embrace contract farming—an innovative aspect addressed in this study. Consequently, the primary objective of the current research is to examine the factors influencing farmers’ intention to adopt contract farming.

### Intention to adopt

The successful adoption of new approaches hinges on the users’ intentions in utilizing these systems. Given the significant investments made by organizations in developing technological platforms for their operations, it becomes crucial to delve into the level of user acceptance and the practical application of emerging approaches and technologies. Organization managers need to be well-versed in understanding and controlling the factors influencing the adoption of these new approaches to effectively achieve their desired goals.

Therefore, the objective of the present study is to establish a model for understanding the intent behind adopting Contract Farming (CF). This is pursued by scrutinizing various behavioral models related to technology adoption, ultimately leading to the widespread acceptance of the Contract Farming approach by users. Table 1 encompasses the behavioral models examined in this study.

**Table 1:** Behavioral models were studied in this study.

Title	Researchers	Variables	Limitations
Theory of Reasoned Action (TRA)	Fishbein and Ajzen, 1975^37^	Attitude toward Behavior, Intention, Subjective Norms	This model overlooked crucial variables, including trust, awareness, perceived ease of use, perceived usefulness, social influence, and extrinsic motivations
Diffusion of Innovation Theory model	Rogers, 1983^38^	Relative advantage, Compatibility, Complexity, Trialability, Observability	This theory fails to take into account the impact of awareness, social influence, extrinsic motivations, and trust on the adoption of an innovation
Technology Acceptance Model (TAM)	Davis et al., 1989^39^	Attitude, Perceived Usefulness, Perceived Ease of Use	This model neglected to consider social influence, extrinsic motivations, trust, and awareness
Theory Of Planned Behavioral (TBP)	Ajzen, 1991^40^	Attitude, Subjective Norm, Perceived Behavioral Control	This model fails to consider important factors such as trust, awareness, extrinsic motivations, social influence, perceived ease of use, perceived usefulness, and other variables that may impact a person’s intention to engage in a behavior
Decomposed Theory of Planned Behavior	Taylor and Todd, 1995^41^	Attitude, Subjective Norms, Perceived Behavioral Control	Trust, extrinsic motivations, social influence, and awareness factors that can significantly influence the intention to adopt a new approach were not taken into account
Dishaw and Strong’s coherent model	Dishaw and Strong, 1999^42^	Intention, Attitude, Perceived Usefulness, Perceived Ease of use, Task Technology Fit, Task Characteristics, Tool Functionality, Tool Experience	In this model, attitude is used as a predictable indicator of behavior, and the intention of adopting to use comes before attitude. Also, in this model, awareness, trust, Extrinsic motivations, and the effect of perceived usefulness and perceived ease of use on attitude are not considered
Technology Acceptance Model 2 (TAM2)	Venkatesh and Davis, 2000^43^	Perceived Usefulness, Perceived Ease of Use	Fundamental variables, including attitude, trust, and awareness, which exert influence on the subjective norm, have been overlooked. Furthermore, the model does not ascertain the factors influencing perceived ease of use, such as extrinsic motivations and social influence
Self-determination theory	Deci and Ryan, 2012^44^	Extrinsic motivation, Intrinsic Motivation, Competence, Relatedness	The consideration of variables, namely trust, awareness, perceived usefulness, perceived ease of use, social influence, and attitude, has not been incorporated
Unified Theory of Acceptance and Use Of Technology (UTAUT)	Venkatesh et al., 2003^45^	Social Influence, Performance expectancy, Effort expectancy, Facilitating conditions, Mediating variables (Gender, Age, Experience, Voluntariness of use)	Important variables such as attitude, trust, perceived usefulness, perceived ease of use, Extrinsic motivations, and awareness have been neglected
(TAM/TPB/IDT)	Mun et al., 2006^46^	Personal Innovativeness, Result Demonstrability, Image, Subjective Norm, Perceived Behavioral Control, Perceived Usefulness, Perceived Ease of use	In this model, awareness, attitude, social influence, Extrinsic motivations, and trust have been ignored
Technology Acceptance Model 3 (TAM3)	Venkatesh and Bala, 2008^47^	Perceived Usefulness, Perceived Ease of use, Mediating variables (Experience, Voluntariness), Image, Subjective Norm, Job Relevance, Output Quality, Result Demonstrability	Important variables such as attitude, trust, Extrinsic motivation, social influence, and awareness have been neglected
Unified Theory of Acceptance and Use of Technology 2 (UTAUT2)	Venkatesh et al., 2012^48^	Social Influence Modulating variables (Gender, Age, Experience), Performance expectancy, Effort expectancy, Facilitating conditions, Hedonic Motivation, Price Value, Habit	Some variables affecting the intention of adopting such as trust, awareness, Extrinsic motivation, Perceived usefulness, Perceived Ease of use, and attitude are not presented in this model
Perceived Characteristics of Innovating Theory (PCIT)	Hameed et al., 2012^49^	Image, Voluntariness, Relative advantage, Compatibility, Ease of use, Demonstrability	Lack of attention to attitude, Perceived Usefulness, awareness, trust, social influence, and Extrinsic variables
Social Cognitive Theory	Rana and Dwivedi, 2015^50^	Behavioral capability, Self-efficacy, Expectations, Self-control, Observational learning, and Reinforcements	This model has overlooked significant variables, including trust, awareness, perceived ease, perceived usefulness, social influence, extrinsic motivations, and attitude, which play crucial roles in the intention of adoption. The Social Cognitive Theory (SCT) model is employed for the evaluation of information technology usage, incorporating constructs such as self-efficacy, performance outcome expectations, anxiety, and personal expectations
Diffusion of Innovation Theory (Extended model)	Rogers, 1995^51^, Dearing and Cox, 2018^52^	Knowledge, persuasion, decision, implementation, and confirmation	This theory does not consider the effect of the variables such as awareness, social influence, and trust. On the other hand, in this model, the adoption of an innovation depends on the mental perception of people and their attitudes
Farmer’s Intention of adopting renewable energy technologies by farmers of Larestan city	Ghorbannejad et al., 2019^53^	Attitude Perceived Usefulness Perceived Ease of Use Trust Awareness Social Influence	In this model, the role of Extrinsic motives is not considered. On the other hand, the direct effect of trust and awareness on the intention to adopt technology has been neglected

References: Research findings.

### Research conceptual framework

In the literature review, it is noted that most behavioral models lack perfection in predicting the performance and integrity of farmers’ intentions to adopt the Contract Farming approach. Furthermore, none of these models demonstrate direct or indirect effects on the key variables of awareness and trust, and there is an absence of exploration into extrinsic motivations. In this study, the Davis Technology Acceptance Model (TAM) was selected as the foundational framework for investigating the intention of farmers in the villages of Markazi Province to adopt the Contract Farming (CF) approach. The key factors, such as attitude, perceived usefulness, and perceived ease of use, are the most important predictors of intention. In the current study, if the components of the Technology Acceptance Model (TAM) had been used alone, it would not have worked as expected. Additionally, other important variables, such as social influence, extrinsic motivation, trust, and knowledge, are added to the TAM model.

The TAM is specifically tailored for modeling the user’s choice regarding a new information system. Thus, it has been most commonly used to predict user acceptance based on perceived usefulness and perceived ease of use in communication technologies^35,54^. In agricultural and food economics, as well as in agricultural extension research, the TAM has emerged as a powerful model for predicting user acceptance and has received considerable empirical support not only in the context of information systems^24,37, 38^ but also in the acceptance of certification systems, precision agriculture, tracking and tracing systems, and renewable energy production^22,39, 45^.

**Figure 1:**
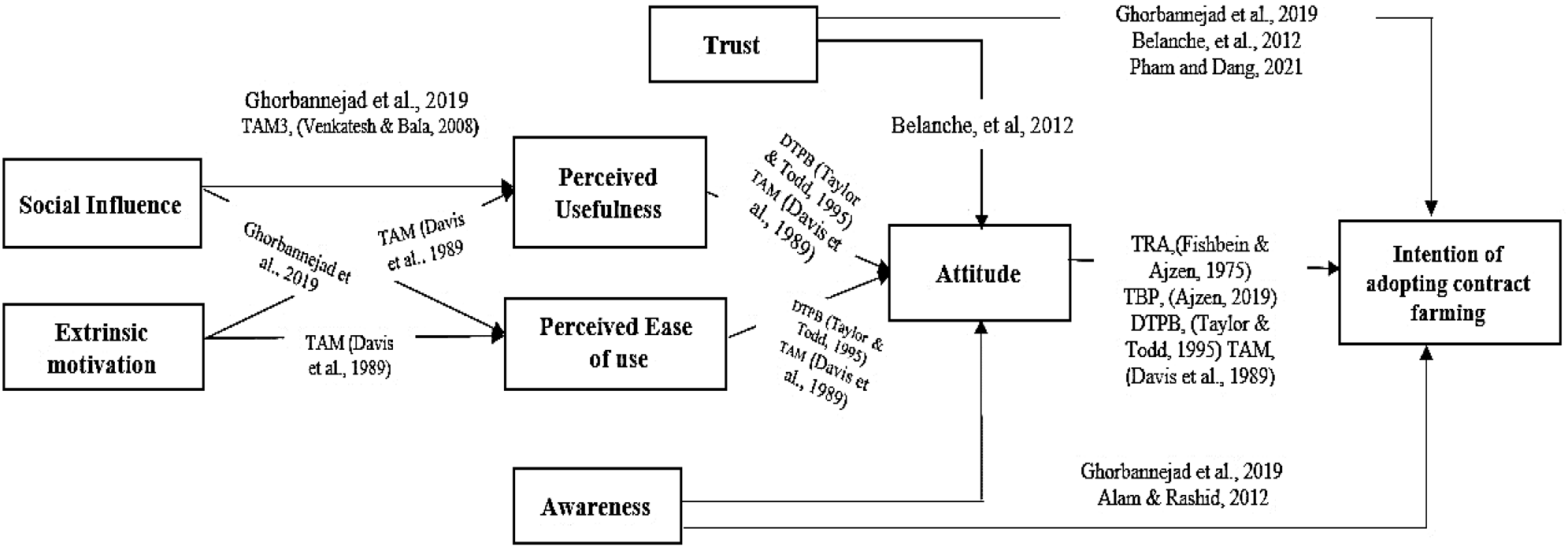
Conceptual framework of the study.

However, the model has not considered important components such as awareness, trust, external motivations, and social influence, which are crucial in the intention of acceptance based on the literature review. By introducing new variables to the TAM model, the model is significantly improved, marking one of the innovative aspects of the current study. Based on the preceding content and literature review, the conceptual model of the study is illustrated in Fig. 1.

The foundational model utilized in this study is based on the Technology Acceptance Model (TAM), with additional variables incorporated through an extensive literature review. The awareness variable, which encompasses the “acquisition of knowledge”, training, and a thorough understanding of a system’s characteristics, significantly influences both behavioral intentions and attitudes. When individuals possess this awareness, it inherently shapes their beliefs and opinions (attitude) regarding whether to engage in a specific action, thus shaping their intention to behave accordingly^55,56^.

Trust, characterized as a “mental state involving the intention to accept risk based on positive expectations,” emanates from individuals having confidence in new technology and methods, grounded in goals or behavior^57^. Trust is the degree of confidence that people have in an approach or technology.

Trust plays a crucial role in the intention to adopt contract farming, representing the level of confidence individuals have in this agricultural method. Especially in contexts marked by risk and uncertainty, trust becomes a fundamental factor influencing technology acceptance. In such environments, individuals are inclined to meticulously assess the situation and its potential consequences, shaping their willingness to embrace new practices like contract farming.

When individuals harbor trust and confidence in the efficacy of contract farming, it cultivates a positive attitude toward its adoption. This positive outlook is rooted in an inherent approval of the method, often developed through firsthand experience. Consequently, this positive attitude exerts a direct and dynamic influence on their behavior, significantly impacting their intention to adopt contract farming practices.

Social influence is measured by the degree to which a person perceives the endorsement of important individuals (e.g., family and friends) regarding their adoption of a particular method. Factors such as social elements and organizational factors, including government support, farmers’ groups, families, and external assistance, contribute to the acceptance of agricultural contracts. Social effect and influence are a kind of value that every person gets through interaction with others. When a person communicates and interacts with others, it affects his understanding of the ease of using a system and also affects the person’s belief that using a certain technology and approach will improve his job performance.

“Extrinsic motivation” serves as a stimulus compelling individuals to engage in a specific activity, driven by external rewards such as facilities, credits, access to inputs, and market entry. Originating from external sources, this form of motivation entails individuals organizing and directing their behavior, anticipating the possibility of receiving rewards. The strong desire instilled by these rewards continues to propel sustained activity until the objectives are achieved^47^.

If there are extrinsic motivators such as credits and facilities, they influence individuals’ perception of the ease and usefulness of adopting a new approach or method. This, in turn, impacts their reactions and feelings, which contribute to their overall attitude. Ultimately, this attitude leads to the intention of acceptance.

Drawing from the research literature, it is predicted that these variables exert both direct and indirect effects on the willingness to accept specific agricultural behaviors. Based on the research literature, it is predicted that these variables have a direct and indirect effect on the intention to accept the behavior. Motivation and social influence, as perceived through usefulness and ease of use, have an impact on and a relationship with attitude^45^. On the other hand, awareness and trust have a direct impact and relationship with the intention to accept, and they also indirectly influence its outcome through attitude.

In the conceptual model of the study, attitude functions as an independent mediating variable affecting the intention to adopt, which is influenced by factors such as ‘Trust,’ ‘Awareness,’ ‘Perceived Usefulness,’ and ‘Perceived Ease of Use.’ These factors indirectly impact the intention to adopt. Furthermore, ‘Trust’ and ‘Awareness’ directly affect the intention to adopt contract farming. Additionally, ‘Social influence’ and ‘Extrinsic motivations’ directly influence ‘Perceived Usefulness’ and ‘Perceived Ease of Use,’ indirectly affecting ‘attitude’ and the intention to adopt contract farming.

## Methodology

### Study area and data collection

**Figure 2:**
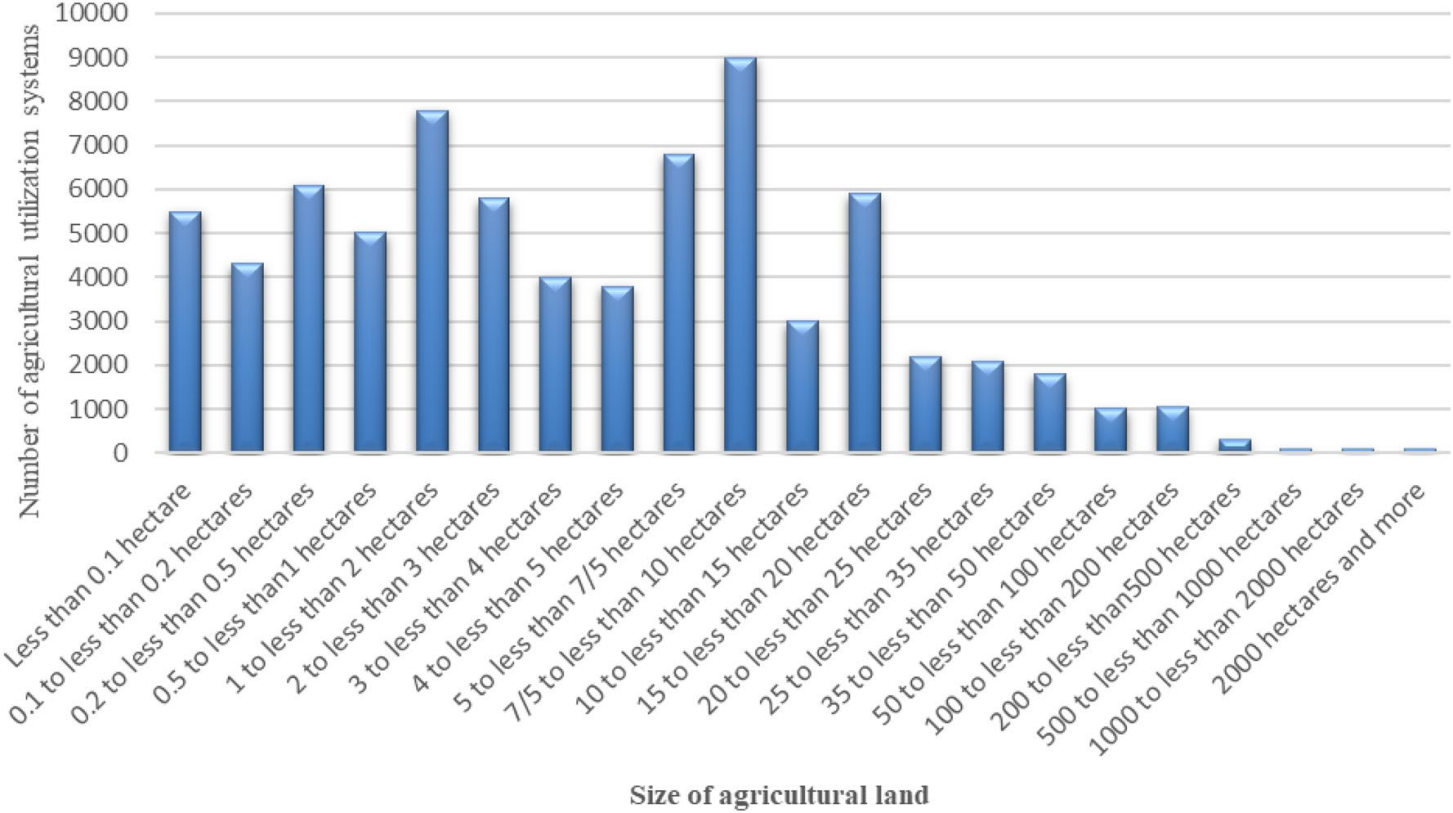
The number of agricultural utilization systems in Markazi Province according to the size of agricultural land^49^.

The research is situated in Markazi Province, where the predominant demographic among agricultural beneficiaries consists of small-scale farmers. According to investigations conducted by the Agriculture Organization of Markazi Province, less than 1% of farmers participate in contract farming—a significantly low percentage, particularly considering the prevalence of small-scale farmers in the province. Figure 2 illustrates the frequency distribution of agricultural utilization systems categorized by classes of agricultural lands in the province.

The census results reveal that the agricultural land in the province spans approximately 553 thousand hectares. These lands are utilized for agriculture and horticulture activities by 67 thousand agricultural operators, with each operation system covering an average of 8.23 hectares. The frequency distribution of agricultural utilization systems among different classes of agricultural land sizes reveals a high relative frequency of systems with limited land, despite the overall quantity of agricultural land at their disposal not being substantial^58^.

The statistical population of the current study is 98,777 farmers from the rural areas of Markazi Province. The sample size in this study was determined to be 383 farmers, considering Karjesi and Morgan’s table.

The tool for collecting data is a researcher-made questionnaire. The study samples were selected using a stratified random sampling. For sampling, first, the cities of Markazi Province were classified based on the predominant field of activity (agriculture and horticulture). From each of these classes, three cities with the most fields of activity were selected. One village was randomly selected from each city and one village was randomly selected from each village (Table 2). It is important to note that the Ethical Committee of Tarbiat Modares University granted approval for the research, and all aspects of the study were conducted in accordance with the relevant guidelines and regulations. Additionally, adherence to the Declaration of Helsinki was ensured throughout the research process.

**Table 2:** Statistical population and selected sample size in Markazi province.

Dominant activity	Selected cities	Selected districts	Selected villages	Statistical populations	Samples
Agriculture	Arak	Aman Abad	Anjdan	86	63
	Khomein	Hamzalu	Imamzadeh Varche	25	18
	Shazand	Astana	Zahir Abad Astana	138	101
Horticulture	Saveh	Salihan	Imamzadeh-Yojan	20	14
	Zarandiya	Khoshkrood	Saidabad	66	48
	Khondab	Khondab	Dehsalman	163	119
Total	6	6	6	498	383

Informed consent was obtained from all participants or their legal guardians. The consent documents were drafted in a language easily comprehensible to participants, allowing them sufficient time for thoughtful consideration of participation. At the outset of the questionnaire, comprehensive explanations were provided regarding the research topic, specifically “contract farming,” and the confidentiality of the results. It was emphasized that the findings would be utilized in designing a behavioral model related to contract farming. Participants were given the choice to participate or decline, and their agreement was signified by endorsing a statement affirming their understanding, satisfaction with answered questions, awareness of the option to withdraw at any time, and voluntary agreement to participate. Farmers constituted the unit of analysis, and the research coordinator played a pivotal role in elucidating issues and addressing potential questions from participants.

### Research tool

A researcher-made questionnaire was used to collect the required data. To measure the intention of adopting contract farming, 5 items have been used, each item with a 5-point Likert scale (1 = very little, 2 = low, 3 = moderate, 4 = high, 5 = very high) was measured. To measure each of the independent study variables (perceived ease of use (6 items), perceived usefulness (9 items), trust in Contract Farming approach (6 items), attitude towards CF (5 items), awareness of CF (6 items), social influence (7 items), Extrinsic motivation (5 items) the five-point Likert scale (1 = very little, 2 = low, 3 = moderate, 4 = high, 5 = very high) was used.

### Validity and reliability determination and data analysis

The content and face validity of the study tools were confirmed using the opinions of subject matter experts, and the reliability of the questionnaire was proven using a pilot test. Therefore, 30 questionnaires were used and completed, and their reliability was confirmed by calculating Cronbach’s alpha coefficient for different parts of the questionnaire using SPSS_25_ Software (Table 3). According to the obtained coefficients, the questionnaire has acceptable reliability, because Cronbach’s alpha value of all parts of the questionnaire was 0.7 or more. To assess the proposed conceptual framework (Fig. 1), validate the model, and investigate the relative impact of independent variables on the dependent variable, structural equation modeling (SEM) and SmartPLS4 Software were used.

**Table 3:** Cronbach’s alpha values of study variables.

ID	Variables	The number of items	Cronbach’s alpha	References
1	Intention of adopting	5	0.72	“TRA^37^”, “TBP^59^”, “DTPB^41^”, “TAM^39^”, “TAM2^43^”, “TAM3^47^”, “Hybrid model (TAM/TPB/IDT)^46^”, “UTAUT1^45^”, “UTAUT2^48^” and “^53^”
2	Perceived Ease of Use	6	0.78	“DTPB^41^”, “TAM/TPB/IDT^46^”, “TAM2^43^”, “TAM3^39^”, “TAM^39^”, “Dishaw and Strong’s coherent model^42^” and “^53^”
3	Perceived Usefulness	9	0.78	“DTPB^41^”, “TAM/TPB/IDT^46^”, “TAM2^43^”, “TAM3^47^”, “TAM^39^”, “Dishaw and Strong’s coherent model^42^” and “^53^”
4	Trust	6	0.71	“^22,24, 35, 37–39, 45, 53–57^”
5	Attitude	5	0.84	“TRA^37^”, “TBP^22,24, 35, 37–45, 45–59^”, “DTPB^41^”, “TAM^39^”, “ DoI model^51,52^”, “Dishaw and Strong’s coherent model^42–53^”
6	Social influence	7	0.77	“UTAUT1^45^”, “UTAUT2^48–53^”, “TAM3^47^”, “DTPB^41^”
7	Awareness	6	0.72	“^53^”
8	Extrinsic motivation	5	0.72	“Self-determination theory^44^”
Total		49	–	

### Results

The descriptive statistics revealed several key findings. The average age of the participants in the study was 50 years. Notably, a majority of the respondents, accounting for 95.6%, were male. In terms of agricultural work experience, participants demonstrated a range of 2 to 55 years, with an average of 17 years. The analysis of the education level variable’s frequency distribution highlighted that the elementary class had the highest occurrence, encompassing 133 individuals (34.7%). On the other hand, the bachelor class and above exhibited the lowest frequency, representing only 11 people (2.9%). Regarding the product buyer variable, an examination of the responses unveiled that a significant portion, specifically 67.1% of the respondents, choose to sell their products to the state company for the storage and marketing of agricultural products.

#### Ranking of the objects of the intention of adopting contract farming

In this study, the mean was employed to rank items related to the adoption of contract farming. As indicated in Table 4, items such as “I will use Contract Farming instead of traditional methods,” “I tend to use Contract Farming to support myself,” and “I anticipate using Contract Farming in the future” achieved the highest ranks in terms of the intention to adopt contract farming.

**Table 4:** Ranking of items of the intention of adopting.

The variable	The items	Mean	SD	Rank
Intention of adopting	IN2. I will use contract farming instead of traditional methods	3.97	0.871	1
	IN4. I want to use contract farming to support my expenses	3.89	0.528	2
	IN3. I expect to use contract farming in the future	3.89	0.572	3
	IN5. I have planned to use farming contracts	3.85	0.538	4
	IN1. I intend to use farming contracts to produce products	3.83	0.582	5

Reference: research findings.

#### Ranking the items of independent variables

Table 5 indicates that, according to the farmers, the three items “By using the farming contract, access to the necessary technology, equipment, and machinery will be easier,” “It is easy to understand the principles and learn the contract farming process,” and “Market access will be increased if farming contracts are used,” respectively, achieved the highest rank among the items in the perceived ease of use category. The overall mean for the perceived ease of use variable was 3.84. In the context of the trust variable, three items have been identified: “I am sure that I have access to the necessary inputs and consulting services if I use contract farming,” “I am sure that I can do the work related to the contracts,” and “I am sure that if I use contract farming, I will have access not only to the current market but also to future markets and even export.” These items have been ranked from first to third among the variables in this category.

**Table 5:** Ranking of independent variables.

Variables	Items	Mean	SD	Rank
Perceived Ease of Use	PE2. Access to the necessary technology, equipment, and machinery will be easier with contract farming	3.97	0.870	1
	PE3. It is easy to learn the principles and process of contract farming	3.89	0.529	2
	PE5. If contract farming is used, access to the market will be increased	3.88	0.571	3
	PE4. It is easy to learn legal issues for the application of contract farming	3.86	0.539	4
	PE6. Contract farming is easy to use	3.84	0.583	5
	PE1. With contract farming; It will be easier to access the needed inputs for crop cultivation	3.36	0.922	6
Total		3.80	0.668	–
Perceived Usefulness	PU3. If contract farming is used, the work will be done more quickly	3.97	0.871	1
	PU2. If contract cultivation is used, the costs related to production and exploitation will be reduced	3.94	0.594	2
	PU4. It is possible to access market information if contract farming is used	3.90	0.572	3
	PU5. Using contract farming increases quantitative productivity	3.89	0.528	4
	PU1. Using contract farming increases product quality	3.85	0.539	5
	PU7. Contract farming has a relative advantage over traditional methods	3.83	0.581	6
	PU8. With coagulation agricultural contracts I will have a fixed income	3.78	0.680	7
	PU6. If contract farming is used, my professional and specialized skills will increase	3.72	0.928	8
	PU9. Contract farming helps me plan for the future planting schedule	3.66	0.708	9
Total		3.84	0.667	–
Trust	TR2. I am sure that if I use contract farming, I will have access to the necessary inputs and consulting services	3.89	0.528	1
	TR3. I am confident that I can do the work related to the contract and documents	3.89	0.573	2
	TR6. I am confident that if I use contract farming, I will have access to the current market, but also future markets and exports	3.85	0.539	3
	TR5. I am sure of the implementation of contract rules and clauses in contract farming	3.83	0.582	4
	TR6. In the case of using contract farming and concluding a contract, I am sure of financing	3.78	0.566	5
	TR1. If I use contract farming, I am sure of selling the products	3.73	0.704	6
Total		3.85	0.582	–
Attitude	AT4. Contract farming provides the basis for creating more jobs in the rural community	3.91	0.922	1
	AT2. Using contract farming will improve the quality of life	3.82	0.583	2
	AT3. Implementation of the contract farming method preserves natural resources and improves the quality of the environment	3.80	0.615	3
	AT5. Households will pay less for the supply of inputs and equipment by concluding contract farming	3.80	0.648	4
	AT1. Contract farming is a suitable alternative to other land use methods	3.78	0.632	5
Total		3.82	0.680	–
Awareness	AW3. I am aware of the advantages of contract farming	3.9	0.527	1
	AW2. I am familiar with the operation of contract farming at the farm level	3.85	0.538	2
	AW4. I am aware of issues related to agricultural contracts	3.84	0.583	3
	AW6. I am familiar with the legal materials and regulations of contract farming	3.56	0.746	4
	AW1. I am familiar with different contract farming models	3.40	0.790	5
	AW5. I am familiar with how to conclude contract farming	2.81	0.783	6
Total		3.56	0.661	–
Social Influence	SI2. Environmentalist unions expect me to use contract farming instead of traditional methods at the farm level	3.97	0.871	1
	SI4. The government expects me to use contract farming instead of the traditional method at the farm level	3.90	0.529	2
	SI5. People whose opinions matter to me prefer that I use a contract farming approach	3.89	0.572	3
	SI3. The rural cooperative expects me to use contract cultivation instead of other exploitation systems	3.85	0.538	4
	SI6. Society expects me to use contract farming instead of the traditional method at the farm level	3.85	0.583	5
	SI1. My family expects me to use contract farming instead of traditional methods at the farm level	3.61	0.794	6
	SI7. Farmers encourage and support other farmers to use farming contracts at the farm level	3.40	0.958	7
Total		3.78	0.692	–
Extrinsic motivation	EM4. The Conditions and time of the contract affect my tendency to coagulate the contract	3.97	0.871	1
	EM5. I will receive government subsidies if I use contract farming,	3.89	0.528	2
	EM3. The type of contract (written or oral) affects my tendency to participate in contract farming	3.89	0.572	3
	EM2. I will benefit from tax exemption if I use contract farming	3.85	0.538	4
	EM1. If I use contract farming, I will benefit from more facilities and credits	3.83	0.582	5
Total		3.88	0.618	–

Reference: research findings.

In the attitude variable, the three items, “Contract Farming provides the basis for creating more jobs in rural society” with an average of 3.91, “The use of Contract Farming will improve the quality of life” with an average of 3.82, and “Implementation of the Contract Farming method preserves natural resources and improves the quality of the environment” with an average of 3.80, respectively, held the highest rank among the items in this variable.

In the awareness variable about contract farming, the items “Awareness of the advantages of contract farming,” “Familiarity with farming contracts,” and “Awareness of farming contract-related issues” respectively attained the highest rank among the variable items.

All items in the social influence variable had an average above 3, contributing to an overall average of 3.78, with the item ‘Environmentalist unions expect me to use contract farming instead of traditional methods at the farm level’ obtaining the highest rank.

In the extrinsic motivation variable, each item attained an average score of approximately 4. This variable demonstrated the highest overall average in comparison to the other variables (see Table 5).

#### Ranking of the influencing factors on the intention of adopting contract farming

After a thorough investigation and ranking of the study variables, the desired variables have been arranged based on the average in Table 6 to facilitate a more precise comparison of the respondents’ views. According to the ranking results, extrinsic motivation holds the highest rank (first position) with an average of 3.88. The overall average of all independent variables was 3.79.

**Table 6:** Ranking of factors affecting the intention of adopting contract farming.

Factors affecting farmers’ intention to adopt contract farming	Mean	Standard deviation	The rank
Extrinsic motivation	3.88	0.618	1
Trust	3.85	0.582	2
Perceived Usefulness	3.84	0.667	3
Attitude	3.82	0.680	4
Perceived Ease of Use	3.80	0.668	5
Social Influence	3.78	0.692	6
Awareness	3.56	0.661	7
Total	3.79	0.654	–

Reference: research findings.

### Correlation coefficients between variables

As presented in Table 7, the results of Pearson’s correlation test revealed a positive and statistically significant relationship among the variables of attitude, trust, awareness, social influence, extrinsic motives, perceived usefulness, perceived ease of use, and the intention of adopting contract farming. This correlation exhibits a noteworthy strength, achieving statistical significance at the one percent error level.

**Table 7:** Correlation matrix between variables.

The first variable	The second variable	Statistical test	Correlation coefficient
Attitude	Intention of adopting	Pearson’s correlation	0.809**
Trust			0.887**
Awareness			0.865**
Social Influence			0.716**
Extrinsic motivation			0.717**
Perceived Ease of Use			0.775**
Perceived Usefulness			0.737**

**Significance at the level of one percent error. Reference: research findings.

### Structural equation modelling

#### The measurement models

In this study, SEM was employed to assess the impact of independent variables (Attitude, Trust, Awareness, Social Influence, Extrinsic motivation, Perceived Ease of Use, and Perceived Usefulness) on individuals’ Intention to adopt contract farming. Before conducting structural analysis, the measurement models of the constructs were evaluated to determine the fit of the data (see Table 8). The baseline measurement model included eight first-order measurement models. Confirmatory factor analysis was employed to evaluate the reliability, convergent validity, and discriminant validity of the constructs. Reliability was assessed using indicators such as the reliability coefficient, composite reliability, and Cronbach’s alpha. The results in Table 8 show that the reliability indicators for all constructs exceeded the threshold value of 0.7 proposed by Vinzi et al.^60^. This finding indicates adequate reliability of the research instrument and its constructs.

**Table 8:** Summary of the measurement model.

Variables	Items	t-value	Standardized factor loadings	Cronbach’s (α)	(CR)	(AVE)
Intention of adopting	IN2	44.57	0.874	0.914	0.914	0.795
	IN3	67.71	0.900			
	IN4	63.22	0.895			
	IN5	68.84	0.897			
Attitude	AT2	46.86	0.859	0.880	0.882	0.736
	AT3	45.71	0.848			
	AT4	63.53	0.883			
	AT5	41.26	0.842			
Trust	TR2	70.96	0.906	0.930	0.931	0.826
	TR3	75.12	0.912			
	TR5	72.28	0.913			
	TR6	66.55	0.913			
Awareness	AW2	54.91	0.882	0.899	0.902	0.767
	AW3	70.05	0.907			
	AW4	45.00	0.870			
	AW6	33.62	0.844			
Perceived Usefulness	PU2	55.11	0.873	0.895	0.897	0.761
	PU3	65.66	0.889			
	PU4	65.13	0.897			
	PU5	34.81	0.828			
Perceived Ease of Use	PE2	59.14	0.884	0.908	0.908	0.783
	PE3	66.73	0.886			
	PE4	55.55	0.878			
	PE5	64.75	0.892			
Social Influence	SI2	64.99	0.888	0.913	0.913	0.793
	SI3	67.86	0.887			
	SI4	75.45	0.897			
	SI5	63.26	0.890			
Extrinsic motivation	EM3	68.26	0.897	0.901	0.903	0.836
	EM4	109.04	0.928			
	EM5	92.15	0.917			

The findings from the AVE index analysis demonstrated the research instrument’s adequate discriminant validity. The values of the index for all constructs in the model exceeded the critical value of 0.5 as suggested by Fornell and Larcker^61^. Furthermore, the Fornell and Larcker criterion matrix revealed that all diagonal values were higher than the correlation coefficient values in the corresponding columns. This discovery, as noted by Hair et al.^62^, further supports the discriminant validity of the research instrument (see Table 9).

**Table 9:** Correlation and square root value of AVE.

Latent variables	AT	AW	EM	IN	PE	PU	SI	TR
AT	0.858							
AW	0.821**	0.876						
EM	0.760**	0.752**	0.914					
IN	0.809**	0.865**	0.718**	0.891				
PE	0.837**	0.783**	0.865**	0.775**	0.885			
PU	0.810**	0.762**	0.865**	0.737**	0.893**	0.872		
SI	0.764**	0.739**	0.849**	0.716**	0.876**	0.878**	0.891	
TR	0.818**	0.888**	0.731**	0.887**	0.781**	0.762**	0.745**	0.909

**P < 0.01.

According to Leguina^63^, in reflective models, the factor loading should be greater than 0.4 for the items to be retained in the model. The results showed that the factor loadings of items IN1, EM1, EM2, PE1, PE6, PU1, PU6, PU7, PU8, PU9, TR1, TR4, AT1, SI1, SI6, SI7, AW1, and AW5 were less than 0.4. Therefore, these items were deleted from the model.

#### Structural model

After confirming the measurement model, the second stage of the analysis involved evaluating the structural model using the PLS-SEM method. This stage aimed to test the proposed hypotheses regarding the relationships between the latent variables of the model. Various indices such as SRMR, exact fit criteria, d_ULS, d_G, and NFI were utilized to assess the model fit. Examination of these indices indicated that the structural model was an acceptable fit for the study of the research hypotheses (Table 10).

**Table 10:** The results of the model fit.

Criteria	Saturated model	Estimated model	Minimum cut-off
SRMR	0.036	0.047	< 0.08
d_ULS	0.656	1.100	p > 0.05
d_G	0.641	0.688	p > 0.05
NFI	0.901	0.898	> 0.90

**Figure 3:**
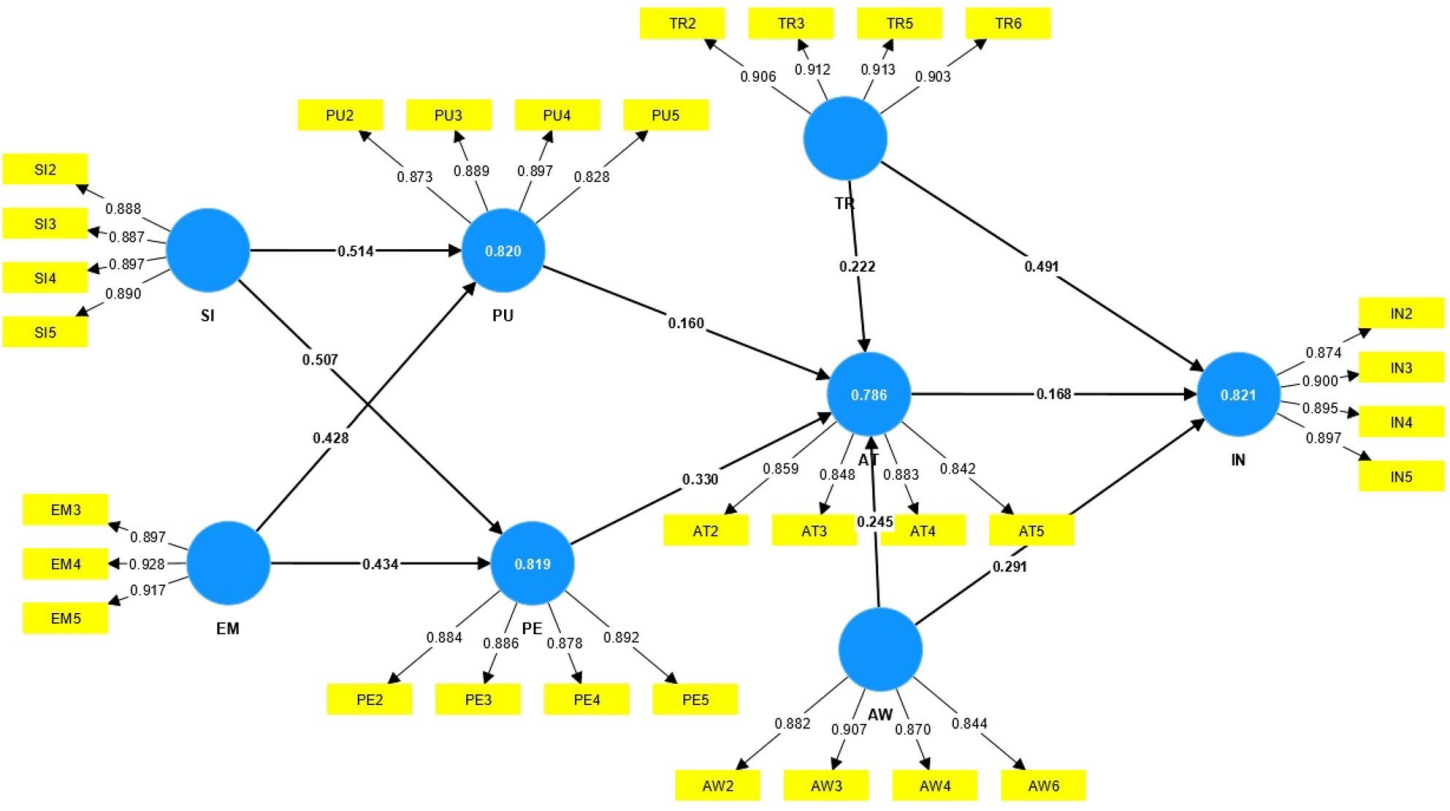
Path coefficient and factor loadings.

**Figure 4:**
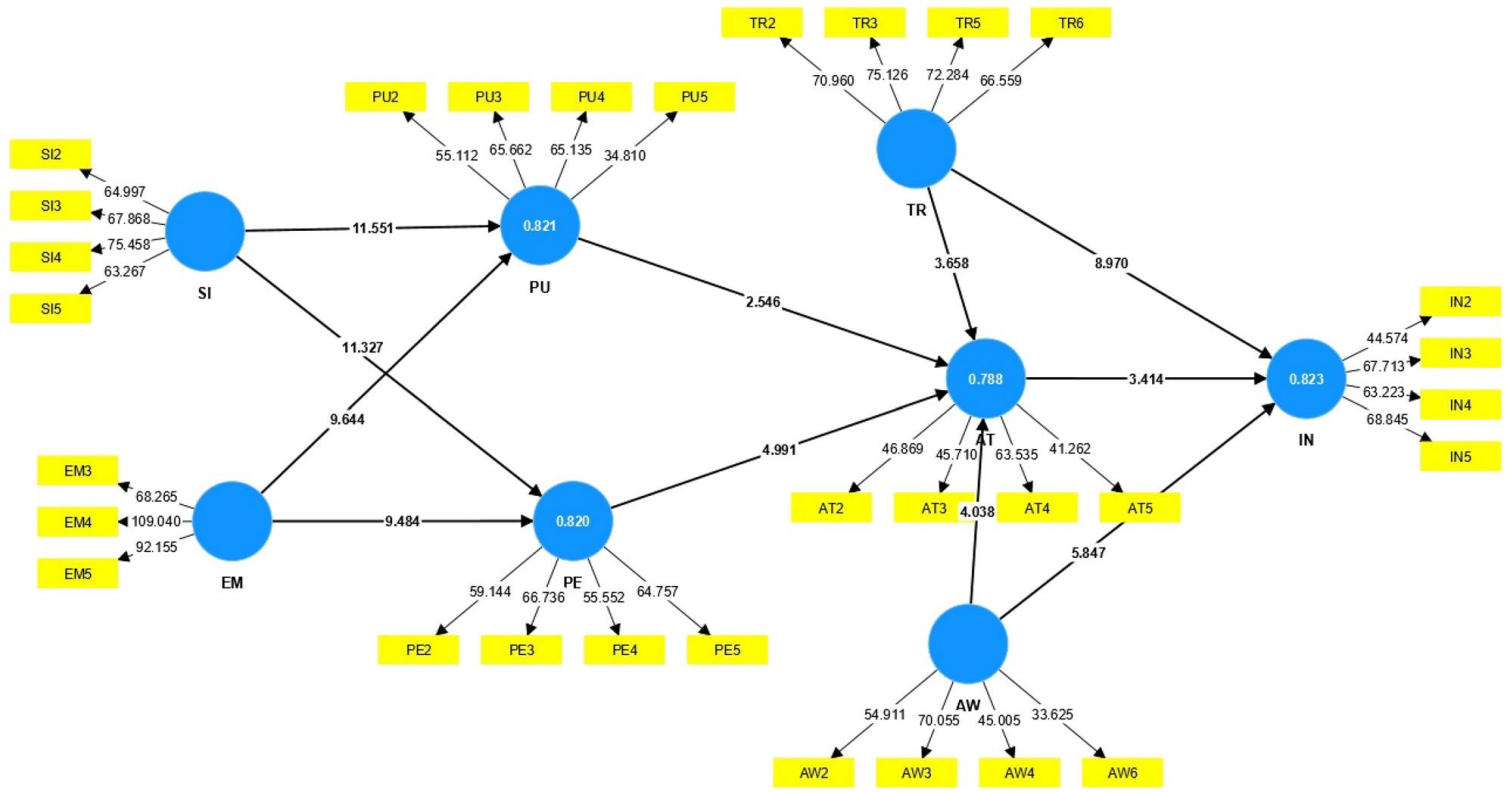
Bootstrapping analysis of the model.

Figures 3 and 4 display the structural model of the research, including the factor loadings and t-values. In this study, the predictive power of the structural model was investigated using the coefficient of determination (R^2^). The R^2^ indicates the proportion of variance in the dependent variable that is explained by the independent variables.

The results showed that the R^2^ for Perceived Usefulness was 0.820, meaning that 82% of the variance in this variable is explained by Social Influence and Extrinsic Motivation. Additionally, the R^2^ for Perceived Ease of Use was calculated to be 0.819, indicating that 81.9% of the variance in this variable is due to Social Influence and Extrinsic Motivation. Furthermore, the R^2^ for Attitude was 0.786, which means that 78.6% of the variance in attitude is predicted by Perceived Usefulness, Perceived Ease of Use, Trust, and Awareness. Finally, the R^2^ for Intention was found to be 0.823, indicating that 82.3% of the variance in this variable is explained by the model’s constructs.

SEM helps researchers calculate the direct and indirect effects of each variable and determine the role of each variable in explaining the dependent variable^35^. Figure 3 shows the total causal effect of studied variables on the intention of adopting. According to Table 11, it presents the impact of various variables on the intention to adopt contract farming. The most substantial effects are associated with three variables: trust (0.528), awareness (0.332), and attitude (0.168). The analysis reveals a positive correlation between social influence and perceptions of ease of use, as well as between social influence and perceptions of usefulness. These perceptions play a pivotal role in shaping individuals’ attitudes. Trust reinforces a favorable attitude towards the method and approach, with individuals relying on trust as a determinant of their attitude towards contract farming. Consequently, this attitude impacts individuals’ behavioral intentions, ultimately influencing their actual behavior.

**Table 11:** The total effect of all independent variables on the intention of adopting contract farming.

Independent variable	Overall effect
Trust	0.528
Awareness	0.332
Attitude	0.168
Perceived ease of use	0.055
Extrinsic motivation	0.034
Social influence	0.042
Perceived usefulness	0.026

When examining “Extrinsic motivation” and the “Perceived Ease of Use,” a positive and statistically significant relationship is observed between motivation and the understanding of usefulness. In other words, motivation and external incentives serve as robust predictors of individuals’ perceptions of ease of use and usefulness. The four variables (ease-of-use perception, perception of usefulness, social influence, and external motivation) contribute directly and indirectly to individuals’ responses, with awareness identified as the primary catalyst in attitude formation.

The results regarding the total effects of the independent variables on the intention of contract farming indicate that among the various predictors of behavioral intention, trust had the most significant overall effect, followed by awareness as the second most influential factor, with attitude ranking third (refer to Table 11). These findings highlight these three components as crucial considerations for future policy-making.

## Discussion

The results reveal that Trust emerges as a significant factor influencing the willingness to adopt contract farming. The farmer’s confidence in the productivity of contract farming correlates with increased risk tolerance^57^. Factors such as expanding market access and timely buyer payments can act as determinants of trust. Trust in business partners enhances the likelihood of entering into crop contracts, and farmers with higher levels of trust are more persuaded towards contract farming than those with lower levels of trust in business relationships^64^. Enhancing knowledge and awareness contributes to a positive attitude towards contract agriculture^66^.

Attitude plays a crucial role as an independent or mediating variable and exhibits a strong and highly significant relationship with adoption intention, essentially driving behavioral intentions. Farmers’ attitudes toward Contract Farming (CF) are influenced by various factors, and effective management by government institutions, private companies, and other influential groups, such as rural cooperatives, can lead to farmers embracing CF, thereby creating value for all stakeholders including producers, consumers, processing industries, exporters, etc. It stands as a viable solution for safeguarding natural resources, preventing environmental crises, managing agricultural risks, and promoting food security. These findings align with the conclusions of Hou et al.^65^, where contract implementation is significantly impacted by farmers’ attitudes and contractual arrangements. Farmers with higher risk tolerance are more inclined to implement contracts^57^. The relationship between attitude, knowledge, and support for the adoption of sustainable agriculture is further supported by the study of Azman et al.^66^.

“Perceived ease of use” has an indirect effect on the intention to adopt through its impact on attitude. This finding is consistent with the results of Vamuloh et al.’s^67^ study. “Extrinsic motivations” influence “attitude” and the “intention to adopt” by affecting “perceived usefulness” and “perceived ease of use”. Individuals organize their behavior by anticipating rewards, and the intensity of the desire for these rewards determines their continued engagement. This observation aligns with Tuan’s^68^ findings. “Social influence” stimulates “attitude” and “intention to adopt” through “perceived usefulness” and “perceived ease of use”. Similar conclusions were drawn in Azmoun et al.’s^52^ study, emphasizing the role of families and friends, the closest people to farmers, in providing moral support and encouragement for sustainable farming practices.

Perceived usefulness has a direct impact on adoption intention through the attitude variable, aligning with the results of Aruna’s^27^ study. All contracts, including the simplest ones, positively impact welfare and productivity measures. This suggests that, when price risk is mitigated through fixed-price contracts, farmers can overcome other constraints on their own, consistent with the findings of Sokchea and Culas^69^.

## Conclusion and suggestions

The main goal of this research is to investigate the factors affecting farmers’ intention to adopt contract farming. Among the factors affecting the intention to adopt contract farming, the variables of attitude, trust, and knowledge had the greatest effect on the intention to adopt contract farming. Attitude is the introduction of behavior. In this context, agricultural extension and education can be highly beneficial by cultivating a positive attitude among farmers toward the adoption of contract farming through educational programs. Providing explanations about the necessity and benefits of using the contract farming approach can further encourage farmers. Also “trust” in the contract farming approach influences adoption intention both directly and indirectly (through attitude). Farmers’ attitudes towards the introduced approaches and methods are mainly motivated by their perceived trust. When users have increased confidence in the new approach, their ability to tolerate risks will be enhanced. It is recommended to establish a unit or center to oversee the implementation of the terms and conditions of the contract. Additionally, it is suggested that investors offer financial, technical, consulting, equipment, and input support to farmers during the production period and ultimately purchase the product at an agreed-upon price. This approach allows farmers to concentrate on planning for the cultivation of the highest quality crop within budget constraints, regardless of other concerns. In addition, awareness of the contract farming approach influences adoption intention, both directly and indirectly (through attitude). A primary obstacle to acceptance is the lack of awareness. Farmers can utilize available information channels to promptly access updates on markets, techniques, epidemics, and weather conditions relevant to crop production. Enhanced knowledge contributes to decreased risks.

More detailed consultation is required to support changes and implement contract farming at the national level. Also, at the local level, there should be encouragement for contract farming with appropriate budget allocations and financial incentives to boost participation in agribusiness. Access to credit is a crucial factor for agribusiness, facilitating the sale and purchase of commodities through contracts. Empowering local authorities (villages, etc.) and farmers involve assisting them in gaining a better understanding of the concept of contract farming, planning at the local level, and identifying situations where the interests of all parties involved, especially farmers, are taken into account can be very fruitful.

In the long term, activities such as providing technical support for the establishment of farmer groups, enhancing negotiation skills, aiding farmers in comprehending the implications of contract farming, conducting market analyses, and managing financial aspects are crucial for empowering farmers. The existence of cooperatives and extension services exerts substantial influence on engagement in contract farming. Consequently, the role of cooperatives and farmer groups should be enhanced in locating input and output markets, augmenting knowledge, and fostering extensive collaboration among members. Government investment in this domain is also advisable.

## Limitations

This study focused exclusively on farmers’ adoption intentions. However, it did not consider the involvement of stakeholders such as companies, planners, and policymakers in designing suitable contracts and implementing contractual programs. Therefore, future research in this field is recommended.

Additionally, given the area of study and the existing knowledge gap in examining the impact and role of extension and education in contract farming, it is suggested that future research explore the effects of extension and education on farmers’ intentions to adopt contract farming.

## Policy and theoretical implications

Based on the findings of the study, there is a positive, significant, and strong relationship between attitude, trust, and awareness of farmers to accept contract farming. Therefore, improving the investment climate to attract and improve the attitude, trust, and awareness of farmers leads to an increase in their intention to adopt contract farming. Therefore, the government should facilitate the investment environment, including private investment in the mentioned fields.

In contract farming, by removing legal restrictions that prevent companies from buying directly from farmers, the government’s role should be to ensure that both parties to the agreement understand and accept the terms. Also, government support for companies and farmers who use this approach, including targeted allocation of subsidies and tax exemptions for the agricultural sector, can be very helpful in this field.

## Data Availability

The datasets generated and/or analyzed during the current study are available in the sciencedb repository, 10.57760/sciencedb.10827.
